# Dietary Supplementation of Ferrous Glycine Chelate Improves Growth Performance of Piglets by Enhancing Serum Immune Antioxidant Properties, Modulating Microbial Structure and Its Metabolic Function in the Early Stage

**DOI:** 10.3389/fvets.2022.876965

**Published:** 2022-04-25

**Authors:** Jiayu Ma, Sujie Liu, Xiangshu Piao, Chunlin Wang, Jian Wang, Yu-sheng Lin, Tzu-ping Hsu, Li Liu

**Affiliations:** ^1^State Key Laboratory of Animal Nutrition, College of Animal Science and Technology, China Agricultural University, Beijing, China; ^2^Shanghai Bestar Biochemical Co. Ltd., Shanghai, China; ^3^Tianjin Zhongsheng Feed Co. Ltd., Tianjin, China

**Keywords:** ferrous glycine chelate, growth performance, antioxidative property, fecal microbiota, piglets

## Abstract

The present research aimed to explore the effect of dietary ferrous glycine chelate supplementation on performance, serum immune-antioxidant parameters, fecal volatile fatty acids, and microbiota in weaned piglets. A total of 80 healthy piglets (weaned at 28 day with an initial weight of 7.43 ± 1.51 kg) were separated into two treatments with five replicates of eight pigs each following a completely randomized block design. The diet was a corn-soybean basal diet with 2,000 mg/kg ferrous glycine chelates (FGC) or not (Ctrl). The serum and fecal samples were collected on days 14 and 28 of the experiment. The results indicated that dietary FGC supplementation improved (*p* < 0.05) the average daily gain and average daily feed intake overall, alleviated (*p* < 0.05) the diarrhea rate of piglets at the early stage, enhanced (*p* < 0.05) the levels of superoxide dismutase and catalase on day 14 and lowered (*p* < 0.05) the MDA level overall. Similarly, the levels of growth hormone and serum iron were increased (*p* < 0.05) in the FGC group. Moreover, dietary FGC supplementation was capable of modulating the microbial community structure of piglets in the early period, increasing (*p* < 0.05) the abundance of short-chain fatty acid-producing bacteria *Tezzerella*, decreasing (*p* < 0.05) the abundance of potentially pathogenic bacteria *Slackia, Olsenella*, and *Prevotella* as well as stimulating (*p* < 0.05) the propanoate and butanoate metabolisms. Briefly, dietary supplemented FGC ameliorates the performance and alleviated the diarrhea of piglets by enhancing antioxidant properties, improving iron transport, up-regulating the growth hormone, modulating the fecal microbiota, and increasing the metabolism function. Therefore, FGC is effective for early iron supplementation and growth of piglets and may be more effective in neonatal piglets.

## Introduction

Iron is one of the trace elements essential for the maintenance of animal life and growth, which is widely involved in oxygen transport, DNA synthesis, electron transfer, oxidative phosphorylation, and other life processes in the body (1). In livestock farming, the addition of exogenous iron in feed has developed over three stages. The first stage is inorganic iron salts represented by ferrous sulfate, ferrous chloride, and ferrous carbonate. However, owing to the low biological utilization of inorganic iron, which often caused the wastage of trace element resources and environmental pollution in actual production, meanwhile, inorganic iron is prone to oxidative stress damage to the organism in the process of absorption, which affects the animal performance (2). The second stage is represented by ferrous citrate and ferrous fumarate as simple organic iron salts. In comparison with inorganic iron salts, simple organic acid iron salts are soluble, absorbable, and highly bioavailable, which have beneficial effects on the growth performance of sows and piglets as well. Nevertheless, the biological potency of different organic iron salts tends to be considerably variable and their weak stability has triggered the development of third-generation iron additives by scholars (3). The third stage is represented by ferrous glycinate, ferric lysine, and ferric methionine, which are chelated with amino acids in the form of ligand bonds to form chelates with a ring structure. The formation of the chelate ring enables the intramolecular charge to become neutral with better chemical stability, which avoids the mutual antagonism between mineral ions with less interference from other factors in the digestive tract to facilitate the absorption and utilization of iron ions by the organism (4). Furthermore, glycine, as the amino acid with the smallest molecular mass, combines with iron to form a complex, which supplies both iron and amino acids to promote the digestion and absorption of iron, thus becoming an effective micronutrient additive required in the feed of farm animals. Studies have demonstrated that ferrous glycinate chelate shows higher bioavailability than ferrous sulfate and with twice the rate of iron absorption in humans, rats, and other animals. Likewise, a study of iron glycinate chelate in the treatment of patients with iron deficiency anemia revealed that iron glycinate chelate has fewer side effects and a greater safety profile than ferrous sulfate (5–7).

Nowadays, iron glycinate chelate has become a hot spot for research on novel iron additives because of its stabilization in the stomach than inorganic iron, which prevents the destructive effect of stomach acid on vitamins and prolongs the shelf life of feeds (4). Limited literature indicated that iron glycinate chelate enhanced the digestive and absorption capacity of animals for improving growth performance (8–10), However, the interactions between iron glycinate chelate and animal intestinal microorganisms and their metabolites have not been elucidated yet.

In the current study, we aim to investigate the effects of iron glycinate chelation on growth performance, serum biochemistry, fecal microbiota, and its metabolites with a piglet model, and combined with functional predictive modeling analysis as well to further clarify microbiota-related mechanisms. Collectively, our study revealed a critical role of iron glycinate chelate on serum biochemistry, microbiota, and its metabolites in piglets, providing a basis for future nutritional interventions in intestinal dysfunction, and the alleviation of diarrhea for piglets.

## Materials and Methods

All procedures of the current study were licensed by the Institutional Animal Care and Use Committee of China Agricultural University (No. AW10601202–1-2, Beijing, China).

### Ferrous Glycine Chelate Products

The commercial ferrous glycine chelate product applied for the current study was supplied by Bestar Biochemicals Co, Ltd (Shanghai, China), which consisted basically of glycine (9%), arginine (0.3%), iron (4.0%), zinc (1.6%), copper (0.4%), and manganese (0.4%), with <10% moisture and citric acid and glucose as carriers.

### Experimental Design, Animal, and Management

A total of 80 ternary crossbred (Duroc × [Landrace × Yorkshire], weaned at day 28, initial weight 7.43 ± 1.51 kg) piglets were separated into two treatments with five replicates of eight pigs (four barrows and four gilts) each with a completely randomized block design. The dietary treatment groups were set as follows: Control group (Ctrl), corn-soybean basal diet; Ferrous glycine chelate group (FGC), a basal diet with 2,000 mg/kg ferrous glycine chelate. The experiment period lasted 28 days. The experimental diets were formulated following the National Research Council Animal Nutrition Board, which developed the Various livestock feeding standards, and usually use the abbreviation (NRC) in livestock (11) to satisfy or exceed the nutritional requirements of piglets, which are presented in Supplementary Table S1. All the experimental diets were powdered.

The experiment was performed at the animal experimental base of China Agricultural University (Hebei, China). The experiment was conducted in a completely enclosed nursery, which was thoroughly cleaned and disinfected prior to piglet transfer and housed in 1.5 × 1.5 m pens. The nursery was furnished with plastic leaky floors, stainless steel adjustable troughs, duckbill drinkers, and a fully automatic environmental control system to monitor the temperature (23–25°C), humidity (65–75%), ventilation intensity, and gas concentrations such as carbon dioxide (0.15%) and ammonia (<20 mg/m^3^) in the house. Piglets were allowed to feed and drink *ad libitum* overall, the nursery was maintained with ventilation and a clean environment, and deworming and vaccination in accordance with the regular management procedures of the base. The whole experiment was strictly complying with the relevant standards of animal welfare of China Agricultural University.

### Growth Performance

The feeding and remaining amount were recorded daily on a pens basis for calculating average daily feed intake (ADFI). The piglets were individually weighed on day 0, day 7, day 14, and day 28 for calculating the average daily gain (ADG), and the feed conversion ratios (FCR) were calculated (ADFI / ADG). Meanwhile, the anal inspection of piglets was carried out individually at 09:00 and 15:00 daily, and the number of piglets with diarrhea was observed and logged to calculate the diarrhea rate of piglets with the following formula:

Diarrhea rate (%) = Number of piglets with diarrhea / (Total number of piglets × Experimental days) × 100%

### Feed/Fecal Collection and Nutritional Analysis

Feed samples of 2 kg were collected from different positions for each treatment group. Two or three pigs per pen were randomly selected, and fresh fecal samples were obtained in a sterile bag by rectal massage on day 14–28. Then stored in a cryogenic refrigerator at −80°C until analyzed. Samples were thawed at 4°C, dried in an oven at 65°C for 72 h. Then fine-grind until the size of 0.38 mm screen can be passed before the chemical analysis. The gross energy (GE), dry matter (DM) ash ether extract (EE), and crude protein (CP) of all feed and fecal samples were analyzed with reference to the method of AOAC (12). Neutral detergent fiber (NDF) and acid detergent fiber (ADF) were determined using a fiber analyzer (ANKOM200 fiber analyzer Ankom, Macedon, USA). The GE was determined by an automatic is metabolic oxygen bomb calorimeter (PARR-6400, Moline, USA). Moreover, an atomic absorption spectrophotometer (Z-5000; Hitachi, Tokyo, Japan) was applied for the determination of chromium in feed and fecal matter according to the methods described by Williams et al. (13). The organic matter (OM) and apparent total tract digestibility (ATTD) were calculated by the equation as follows:

OM (%) = 1-ash (DM-basis) × 100%

ATTD (%) =1-(Cr feed × Nutrient feces) / (Cr feces × Nutrient feed) × 100%

### Serum Collection and Determination

On day 7, day 14, and day 28, respectively, approximately 10 ml of blood was collected from the anterior vena cava of 10 piglets with nearly average body weight (one barrow and one gilt per pen) per treatment using vacuum blood collection tubes, left for 30 min at room temperature, centrifuged at 3,000 g for 10 min at 4°C to prepare serum, then stored at −20°C until determination.

The growth hormone content in serum was analyzed using a multi-tube radioimmunoassay counter (BFM-96, Zongcheng Electro-Mechanical Technology, Guangzhou, China). Serum immunoglobulin A (IgA), immunoglobulin G (IgG), immunoglobulin M (IgM), inflammatory factors interleukin-6 (IL-6), interleukin-8 (IL-8), interleukin-10 (IL- 10), tumor necrosis factor-α (TNF-α), gamma-interferon (IFN-γ), D-lactate (D-LA), glucocorticoid, adrenocorticotropic hormone (ACTH), epinephrine, xanthine oxidase (XOD), serum iron, and hepcidin activity were determined using Multiskan Ascent fully automated enzyme marker (Thermo Scientific, Waltham, USA) and enzyme linked immunosorbent assay (ELISA) kits.

Total cholesterol (TC), total triglycerides (TG), low-density lipoprotein (LDL), high-density lipoprotein (HDL), glucose, total protein (TP), albumin, non-esterified fatty acids (NEFA), total iron-binding capacity (TIBC), uric acid (UA), blood urea nitrogen (BUN), alanine aminotransferase (ALT), aspartate aminotransferase (AST), alkaline phosphatase (AKP), lactate dehydrogenase (LDH), malondialdehyde (MDA), superoxide dismutase (SOD), glutathione peroxidase (GSH-Px), catalase (CAT), and total antioxidant capacity (T-AOC) levels were measured using a fully automated biochemical instrument (CLS880, Jiangsu Zecheng Biotechnology, Wuxi, China). All kits were sourced from Nanjing Jiancheng Institute of Biological Engineering (Nanjing, China), and all assay procedures were conducted strictly in accordance with the instructions of the manufacturer.

### Volatile Fatty Acid

The fecal samples were thawed at 4°C and mixed homogeneously. Approximately 0.5 g of the sample was placed in a 10 ml polypropylene tube and 8 ml of ultrapure water was added, then centrifuged at 12,000 g for 10 min at 4°C after sonication for 30 min in an ice-water bath (mixing per 10 min). The supernatant was extracted and diluted 50 times with ultrapure water, filtered through a 0.22 mm membrane, and added to the injection vial. The analysis was carried out using ICS-3000 ion chromatography (ICS-3000, Thermo Scientific, USA), the external standard solution containing eight organic acids was obtained from Sigma-Aldrich (Saint Louis, USA). A variety of organic acids were separated by an AS11 analytical column (250 × 4 mm) and an AG11 guard column (50 × 2 mm) under the mobile phase elution conditions: potassium hydroxide gradient, 0–5 min, 0.8–1.5 mM; 5–10 min, 1.5–2.5 mM; 10–15 min, 2.5 mM, 1 ml/min flow rate.

### 16s RRNA Gene Sequencing and Analysis

The fecal samples were removed from the −80°C refrigerator and analyzed for 16 S rRNA sequencing. Fecal DNA was extracted using the Qiagen QIAMP DNA stool extraction kit (QIAGEN, Frankfurt, Germany), according to the instructions. The concentration and purity of DNA were monitored by 1% agar gel electrophoresis. PCR amplification of the 16 S rRNA gene in the variable region of bacterial V3-V4 was conducted following the universal primers 338F (5'-A CTCCTACGGGAGGCAGCAG-3') and 806R (5'-GGA CTACHVGGGTWTCTAAT-3').

The protocol for the 20 μl PCR reaction system is as follows: 5 × FastPfu buffer, 4 μl, 2.5 mM dNTPs, 2 μl, Forward primer(5 μM), 0.8 μl, Reverse primer (5 μM), FastPfu polymerase, 0.4 μl, bovine serum albumin, 0.2 μl, template DNA, 10 ng, supplemented with double-distilled H_2_O to 20 μl. PCR amplification procedure: 95°C pre-denaturation for 3min, 30 cycles (95°C denaturation for 30 s, 55°C annealing for 30 s, 72°C extension for 45 s), followed by 72°C stable extensions for 10 min (PCR instrument: ABI GeneAmp^®^ 9700 model, Foster City, USA; PCR efficiency: 96–103%).

PCR products were detected with 2% agarose gel electrophoresis and mixed, then stocked using the kit Axyprep DNA Gel Extraction Kit (Axygen Biosciences, Tewksbury, USA). Sequence libraries suitable for sequencing by Illumina (San Diego, USA) were prepared according to the operating instructions of the NEB Next Ultra DNA Library Prep Kit. Library quality was evaluated using a Qubit fluorescence spectrophotometer (Thermo Scientific, Waltham, USA) and an Agilent Biochemical Analysis 2100 system. Lastly, the libraries were sequenced using the Illumina Miseq platform and 250bp double-end reading sequences were generated. The double-end read sequences were assigned to the respective samples based on the different marker sequences and sequence splicing was performed using FLASH software (14). The UPARSE software (http://drive5.com/uparse/, version 7.1) based on the UPARSE-OUT (15) and UPARSE- OTUref algorithms were applied to cluster sequence reads into operational classification units (OTUs) at 97% identity level (16) and Taxonomic analysis of OTU representative sequences using RDP classifier (http://rdp.cme.msu.edu/, version 2.2) based on Bayesian algorithm, then identified taxonomy was aligned using the Silva 16S rRNA database (v138, http://www.arb-silva.de) with 70% confidence threshold (17). The original contributions presented in this study are included in the article/Supplementary Material, further inquiries can be directed to the corresponding author/s. The microbial dataset has been deposited on NCBI with accession code PRJNA785430 (https://www.ncbi.nlm.nih.gov/bioproject/PRJNA785430).

### Statistical and Bioinformatic Analysis

All data were preliminarily organized by Excel software (Microsoft, Redmond, USA) and statistically analyzed using an unpaired Student's *t*-test by SAS 9.2 (SAS Institute, NC, USA) excluding the diarrhea rate, which was analyzed using the chi-square test. The serum indicators were analyzed on an individual basis and the growth performance, apparent digestibility of nutrients, diarrhea rate, and volatile fatty acids were analyzed on a pen basis. Means and standard errors of mean (SEM) were calculated using the Least-squares means (LSMEANS) method. Statistically, significant differences were considered when *p* < 0.05 and statistical trend when *p* < 0.10.

α-Diversity, a measure of the richness, evenness, and diversity of the bacterial community, was evaluated by calculating the sobs, Shannon, Simpson, ace, chao, and phylogenetic diversity (pd) indices using the mothur (version v.1.30.2 https://mothur.org/wiki/calculators/). Principal coordinates analysis (PCoA), one comparing similarities or differences in the composition of sample communities between treatments, was estimated by R software (version 3.3.1) based on the Bray-Curtis distance matrix algorithm and analysis of similarities (ANOSIM). Circos reflected not only the proportion of dominant species composition in each (or group) sample but also the proportion of distribution of each dominant species in different samples (subgroups), which was plotted using Circos-0.67-7 (http://circos.ca/). A Welch's *t*-test with FDR multiple test correction was used for statistical difference analysis between treatments at the family, phylum, and genus levels, respectively. The LEfSe analysis, based on the non-parametric factorial Kruskal–Wallis (KW) sum-rank test and Wilcoxon rank-sum test, was used to estimate features with significant abundance differences and to identify taxa that differed significantly from the abundance and only taxa with LDA score>3 was displayed. Spearman's correlation heatmap illustrated the correlation between growth traits and genus levels. The Clusters of Orthologous Groups (COG) functional classification and Kyoto Encyclopedia of Genes and Genomes (KEGG) metabolic function of fecal microbiota was predicted using PICRUST2 and plotted using Prism 8.0 (GraphPad, La Jolla, CA).

## Results

### Growth Performance

As displayed in Table 1, supplementation of FGC increased (*p* < 0.05) the ADG for piglets at day 0–7, day 15–28 and overall, respectively. The higher (*p* < 0.05) ADFI was observed for piglets supplemented with FGC at day 15–28. Also, the diarrhea rate of piglets receiving FGC supplementation declined (*p* < 0.05) from day 0–7.

**Table 1:** Growth performance of piglets as affected by dietary ferrous glycine chelate supplementation.

**Items**	**Ctrl ^**1**^**	**FGC**	**SEM ^**2**^**	***p*-Value**
Initial weight	7.43	7.43	0.01	0.62
D 0-D 7
ADG ^3^, g	**227.39** ^**b**^	**248.37** ^**a**^	**4.59**	**0.03**
ADFI, g	372.42	385.73	11.89	0.47
FCR	1.64	1.55	0.07	0.38
Diarrhea rate, %	**2.86** ^**a**^	**0.36** ^**b**^	**0.56**	**0.02**
D 8-D 14
ADG, g	313.25	325.46	11.22	0.48
ADFI, g	579.36	596.22	6.30	0.13
FCR	1.85	1.84	0.07	0.88
Diarrhea rate, %	1.79	2.14	0.62	0.71
D 0-D 14
ADG, g	270.32	286.91	6.06	0.13
ADFI, g	475.89	490.98	5.48	0.12
FCR	1.76	1.71	0.03	0.35
Diarrhea rate, %	2.32	1.25	0.43	0.08
D 15-D 28
ADG, g	**386.79** ^**b**^	**464.31** ^**a**^	**18.59**	**0.04**
ADFI, g	**719.5** ^**b**^	**807.17** ^**a**^	**20.33**	**0.05**
FCR	1.86	1.74	0.06	0.22
Diarrhea rate, %	3.27	1.07	1.07	0.22
D 0-D 28
ADG, g	**329.37** ^**b**^	**375.61** ^**a**^	**10.86**	**0.04**
ADFI, g	597.7	649.08	18.06	0.11
FCR	1.81	1.72	0.04	0.23
Diarrhea rate, %	2.80	1.16	0.50	0.09

a, b *different superscripts within a row mean significant difference (p < 0.05)*.

1 *Ctrl, corn-soybean basal diet; FGC, corn-soybean basal diet with 2,000 mg/kg ferrous glycine chelate*.

2 *SEM is the standard error of the mean*.

3 *ADG, average daily gain; ADFI, average daily feed intake; FCR, feed conversion ratios*.

*The bold values indicate significant differences*.

### Immune, Inflammatory Characteristics, and Serum Metabolites

As illustrated in Supplementary Figure S1, neither serum immune parameters nor inflammatory factors were observed to have significant differences between the treatment and control groups on days 7, 14, and 28.

**Figure 1:**
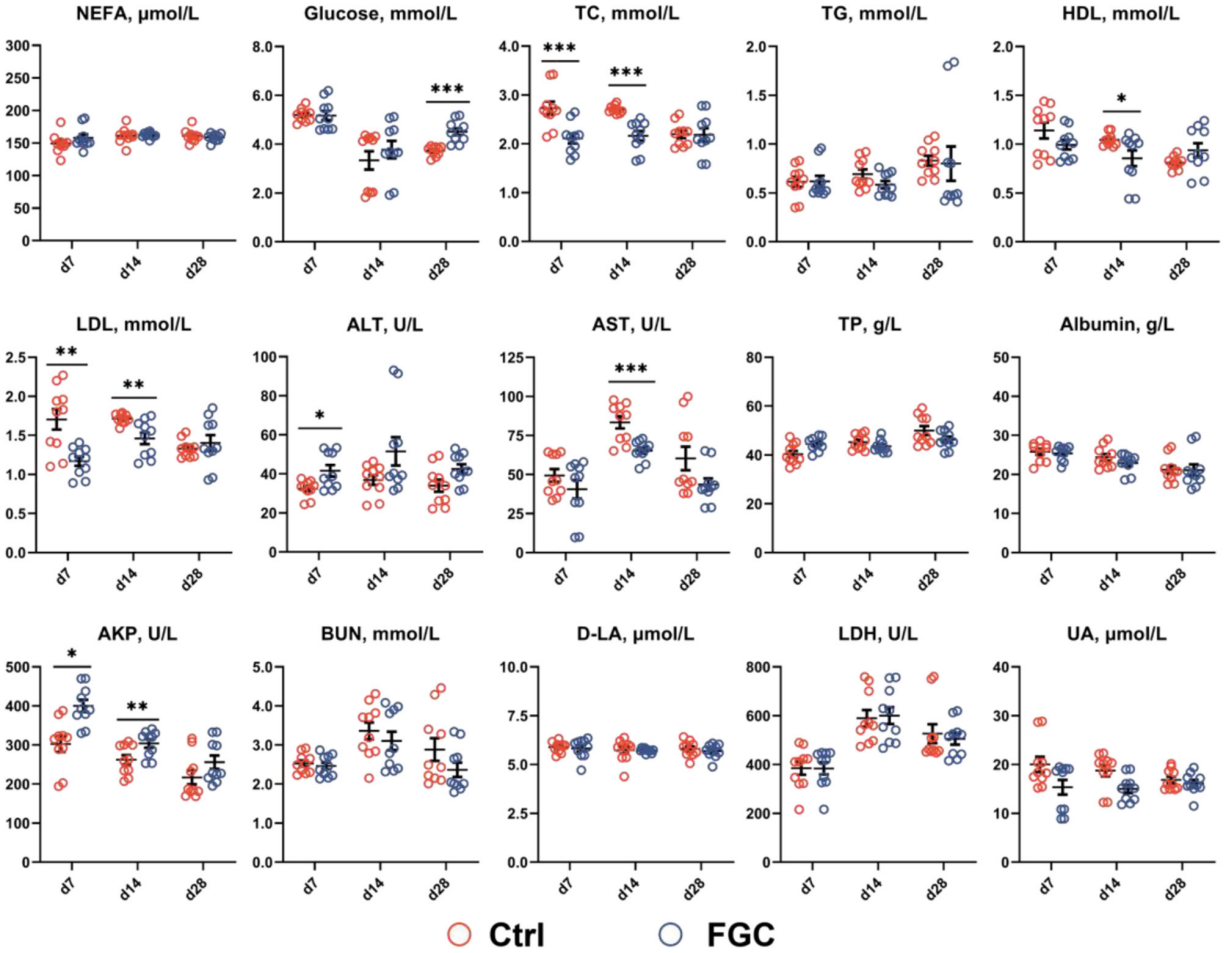
Serum metabolites of 7d, 14d and 28d-piglets as affected by dietary ferrous glycine chelate supplementation. NEFA, non-esterified fatty acids; TC, total cholesterol; TG, total triglycerides; HDL, high density lipoprotein; LDL, low density lipoprotein; ALT, alanine aminotransferase; AST, aspartate aminotransferase; TP, total protein; AKP, alkaline phosphatase; BUN, blood urea nitrogen; D-LA, D-lactate; LDH, lactate dehydrogenase; UA, uric acid. Ctrl, corn-soybean basal diet; FGC, corn-soybean basal diet with 2,000 mg/kg ferrous glycine chelate. Data were shown as means ± SEM. *p < 0.05; **p < 0.01; ***p < 0.001. *N* = 10.

As illustrated in Figure 1, on day 7, piglets supplemented with FGC exhibited the serum TC and LDL with a lower level (*p* < 0.05) and the serum ALT and AKP with a higher content (*p* < 0.05). On day 14, the serum TC, HDL, LDL, and AST levels were reduced (*p* < 0.05) and serum AKP level was elevated (*p* < 0.05) in piglets receiving FGC supplementation. On day 28, a higher (*p* < 0.05) serum glucose content was noticed in the FGC group. Nevertheless, with regard to other serum metabolites, there were no significant differences between the treatment and control groups.

### Antioxidant Capacity and Hormones

**Figure 2:**
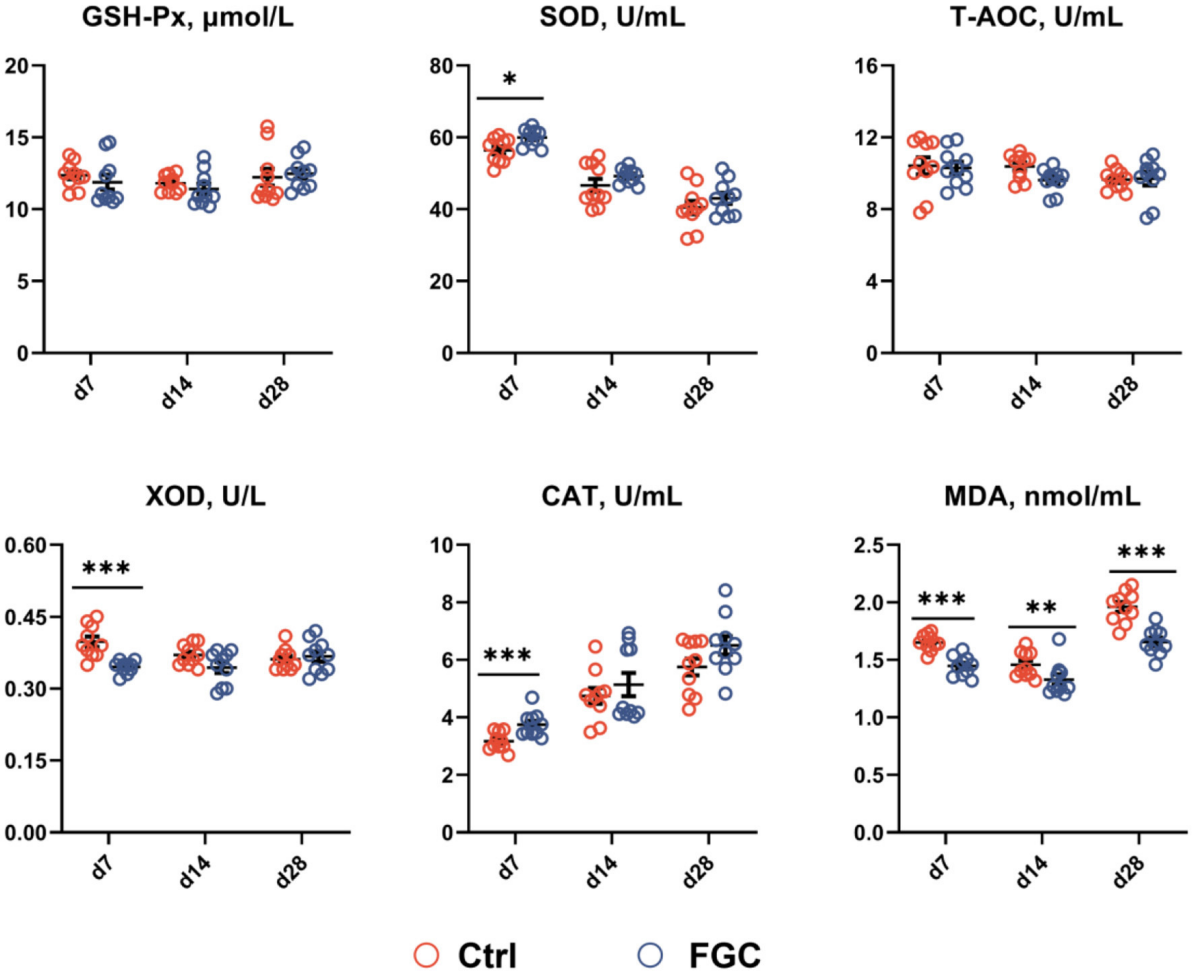
Serum antioxidant capacity of 7d, 14d and 28d-piglets as affected by dietary ferrous glycine chelate supplementation. GSH-Px, glutathione peroxidase; SOD, superoxide dismutase, T-AOC, total antioxidant capacity; XOD, xanthine oxidase; CAT, catalase; MDA, Malondialdehyde. Ctrl, corn-soybean basal diet; FGC, corn-soybean basal diet with 2,000 mg/kg ferrous glycine chelate. Data were shown as means ± SEM. *p < 0.05; **p < 0.01; ***p < 0.001. *N* = 10.

As illustrated in Figure 2, piglets supplemented with FGC enhanced (*p* < 0.05) the SOD and CAT contents and lowered (*p* < 0.05) the XOD level. Moreover, dietary supplementation of FGC significantly decreased (*p* < 0.05) the MDA level on days 7, 14, and 28, respectively. No impact on serum GSH-Px and T-AOC by FGC supplementation.

**Figure 3:**
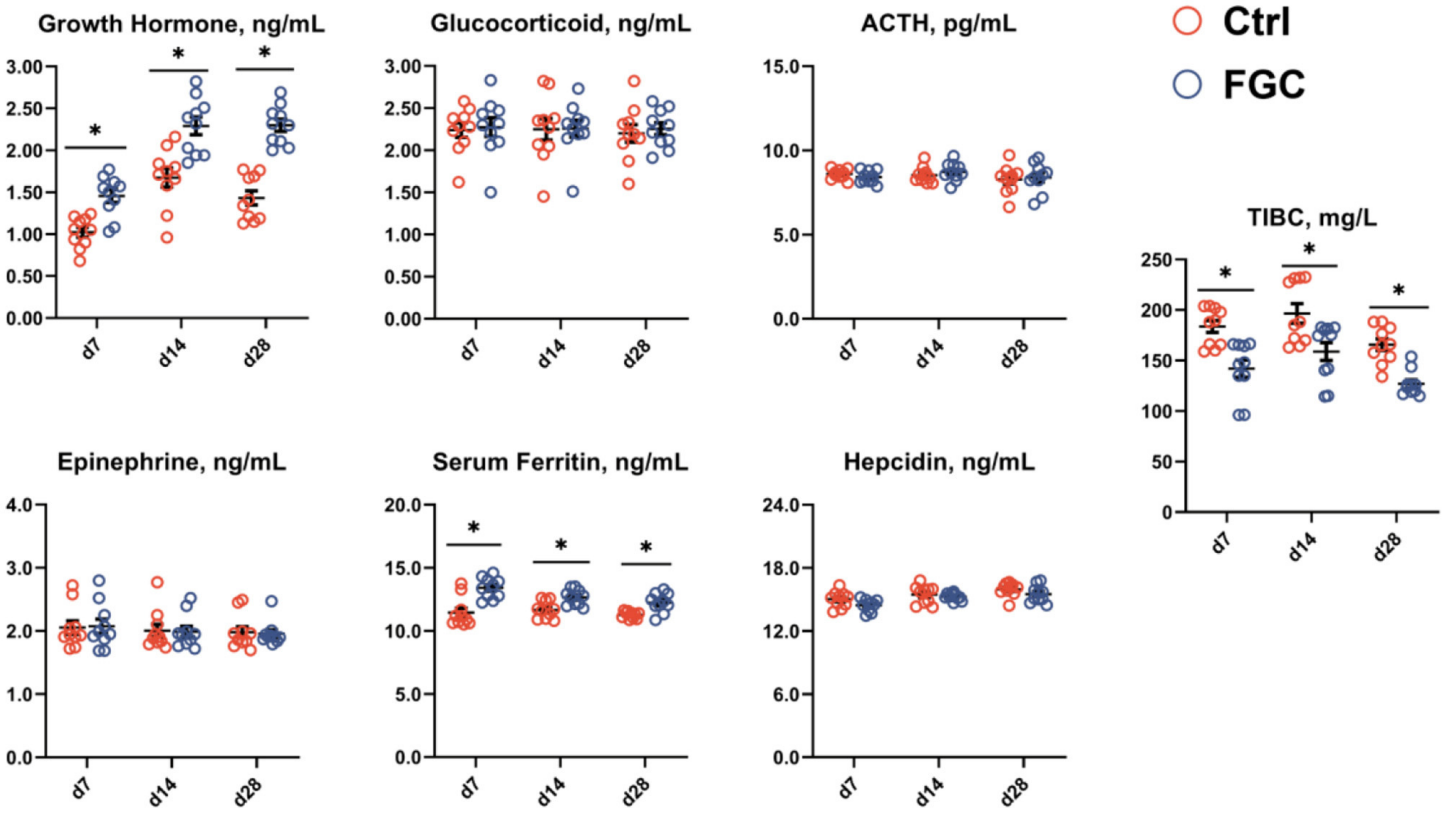
Serum hormones and iron-binding capacity of 7d, 14d and 28d-piglets as affected by dietary ferrous glycine chelate supplementation. ACTH, adrenocorticotropic hormone; TIBC, total iron binding capacity. Ctrl, corn-soybean basal diet; FGC, corn-soybean basal diet with 2,000 mg/kg ferrous glycine chelate. Data were shown as means ± SEM. *p < 0.05. *N* = 10.

As illustrated in Figure 3, dietary supplementation of FGC significantly enhanced (*p* < 0.05) the serum growth hormone and serum iron levels while decreasing (*p* < 0.05) the serum TIBC on days 7, 14, and 28, respectively. Also, FGC supplementation had no effect on serum levels of glucocorticoids, ACTH, epinephrine, and hepcidin.

### Apparent Nutrient Digestibility

As shown in Table 2, piglets fed with FGC improved (*p* < 0.05) the apparent total tract digestibility of EE on day 14 and acid detergent fiber on day 28. No other significant differences were noticed between Ctrl and FGC groups.

**Table 2:** ATTD of piglets as affected by dietary FGC supplementation, %.

**Items**	**Ctrl^**1**^**	**FGC**	**SEM ^**2**^**	***p*-Value**
D14				
Dry matter	81.28	80.98	0.58	0.73
Organic matter	84.83	84.53	0.57	0.73
Crude protein	73.63	73.18	0.92	0.75
Ether extract	62.08^b^	65.44^a^	0.70	0.03
Gross energy	80.50	80.02	0.52	0.55
Neutral detergent fiber	48.70	53.42	2.96	0.32
Acid detergent fiber	46.84	51.68	2.73	0.28
D28				
Dry matter	83.29	82.32	0.62	0.33
Organic matter	86.48	85.78	0.51	0.39
Crude protein	75.66	74.63	1.32	0.61
Ether extract	67.52	68.68	2.78	0.78
Gross energy	82.64	81.91	0.78	0.55
Neutral detergent fiber	54.58	57.83	2.14	0.34
Acid detergent fiber	52.58^b^	58.02^a^	1.14	0.03

a, b * different superscripts within a row mean significant difference (p < 0.05). N = 5*.

1 *Ctrl, corn-soybean basal diet; FGC, corn-soybean basal diet with 2,000 mg/kg ferrous glycine chelate*.

2 *SEM is the standard error of the mean*.

### Fecal Microbial Sequencing Data and Diversity Indices

On days 14 and 28 of the experiment, 15 weaned piglet fecal samples were collected, and 16SrRNA sequencing was performed using the IlluminaHiseq high-throughput sequencing platform. There were a total of 2,276,531 high-quality raw-reads obtained. All samples were randomly flattened at the minimum sample sequence for the avoidance of errors due to differences in sequencing depth. Based on 97% sequence similarity, a total of 797 OTUs were identified and classified into 13 phyla, 20 classes, 44 orders, 77 families, 187 genera, and 370 species in the 15 piglet fecal samples on day 14 of the experiment, and a total of 831 OTUs were identified and classified into 13 phylum, 21 classes, 45 orders, 76 families, 201 genera, and 389 species in the 15 piglet fecal samples on the 28th day of the experiment after comparison with the Silva database.

**Figure 4:**
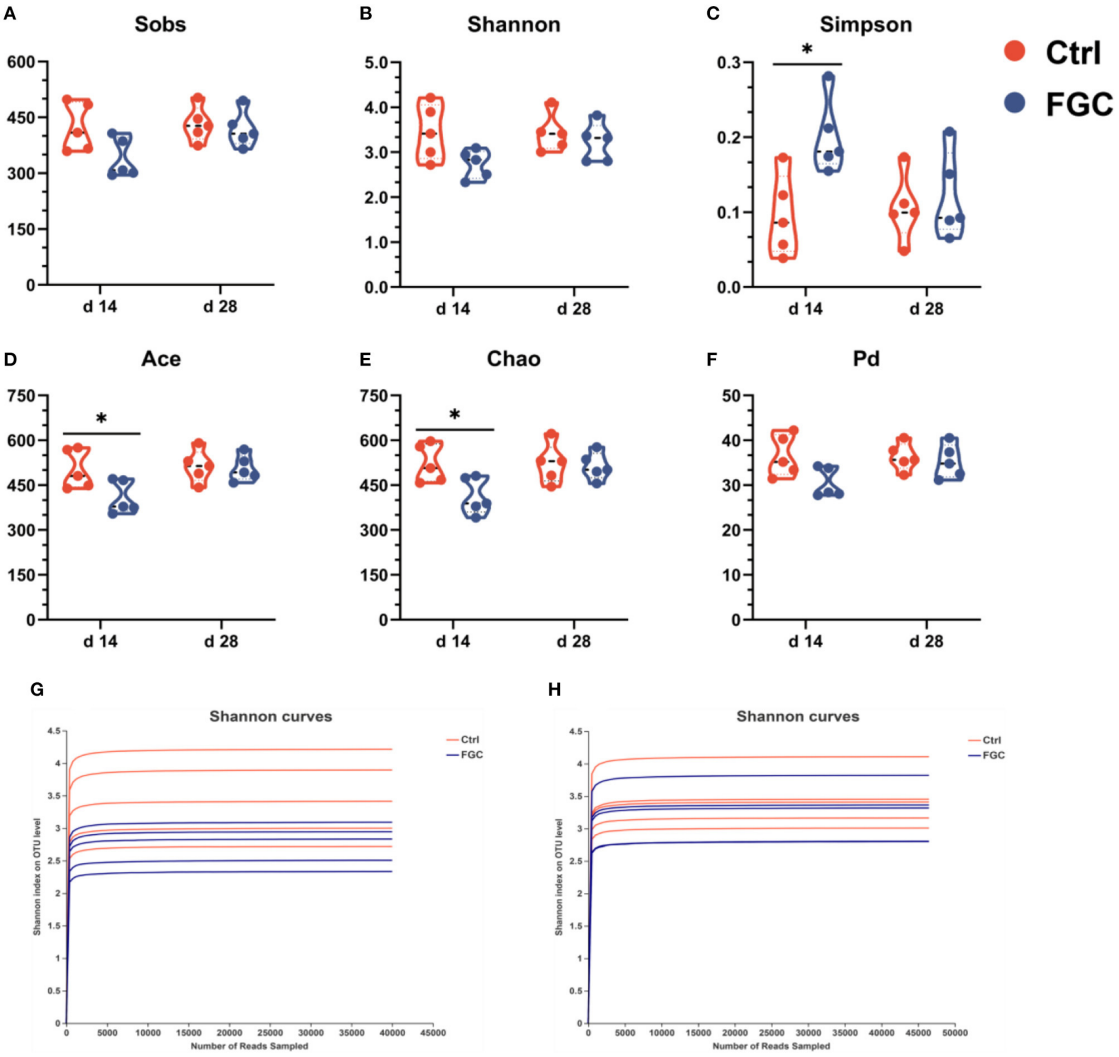
Fecal microbial α-diversity and rarefaction curves at OTU level. **(A)** Sobs index. **(B)** Shannon index. **(C)** Simpson index. **(D)** Ace index. **(E)** Chao index. **(F)** phylogenetic diversity index. **(G)** Shannon curves at d 14. **(H)** Shannon curves at d 28. Ctrl, corn-soybean basal diet; FGC, corn-soybean basal diet with 2,000 mg/kg ferrous glycine chelate. Data were shown as means ± SEM. *p < 0.05. *N* = 5.

As illustrated in Figure 4, the analysis of dilution curves based on the Shannon index indicated a dramatically rising diversity index for all samples (Figures 4G,H), followed by a gradually increasing trend and finally saturation, suggesting that the sequencing data volume was sufficient. Also, dietary supplementation of FGC increased (*p* < 0.05) the microbial diversity indices Ace and Chao (Figures 4D,E) and lowered (*p* < 0.05) the Simpson index (Figure 4C) on day 14. Nevertheless, no differences in microbial diversity indices between the treatment and control groups were observed on day 28.

### Fecal Microbial Composition and β-Diversity Analysis

**Figure 5:**
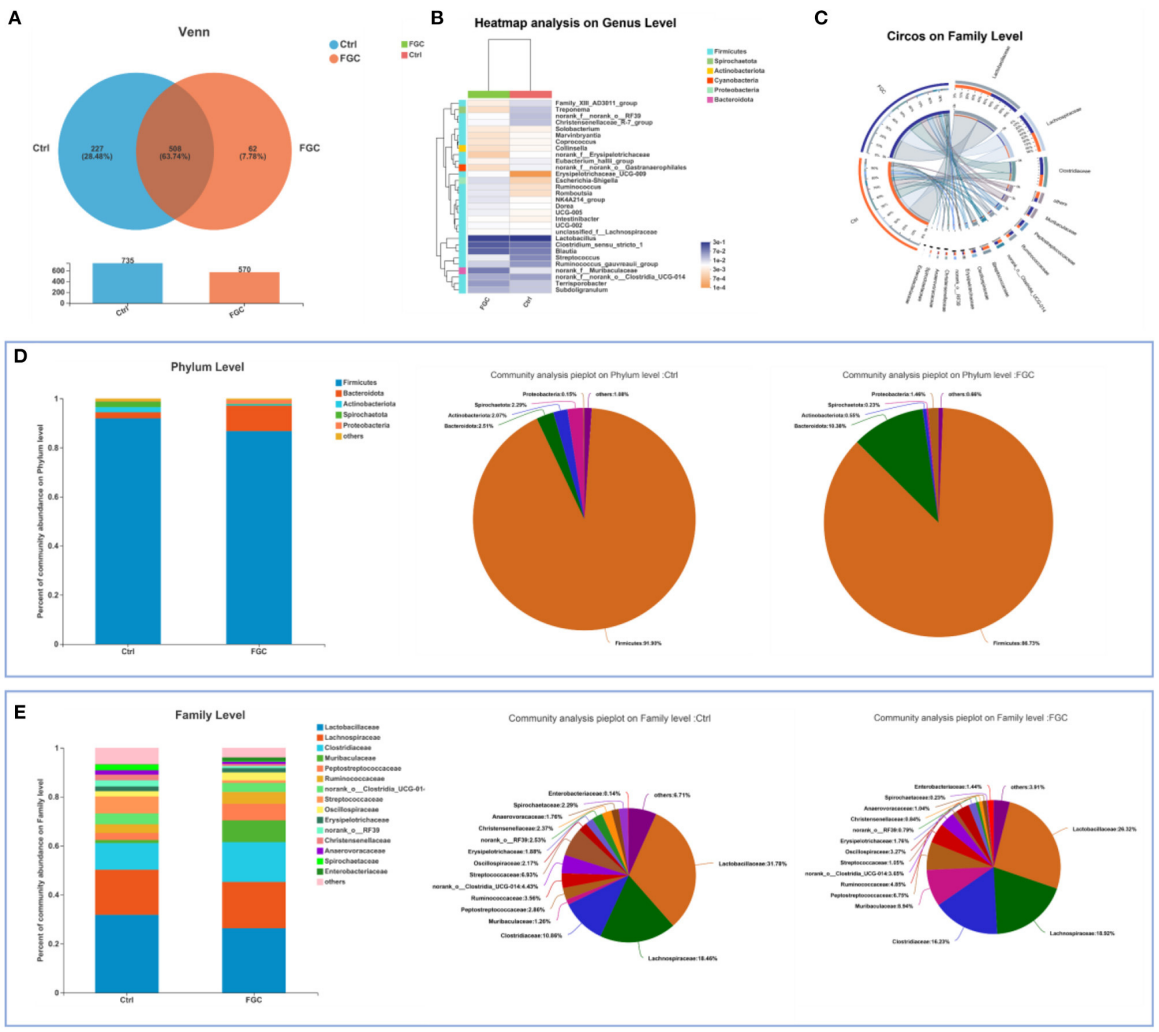
Overview of fecal microbial composition of 14d-piglets. **(A)** Venn diagram. **(B)** Heatmap at genus level. **(C)** Circos diagram at family level. **(D)** Barplot and pieplot at phylum level. **(E)** Barplot and pieplot diagram at family level. Ctrl, corn-soybean basal diet; FGC, corn-soybean basal diet with 2,000 mg/kg ferrous glycine chelate. *N* = 5.

**Figure 6:**
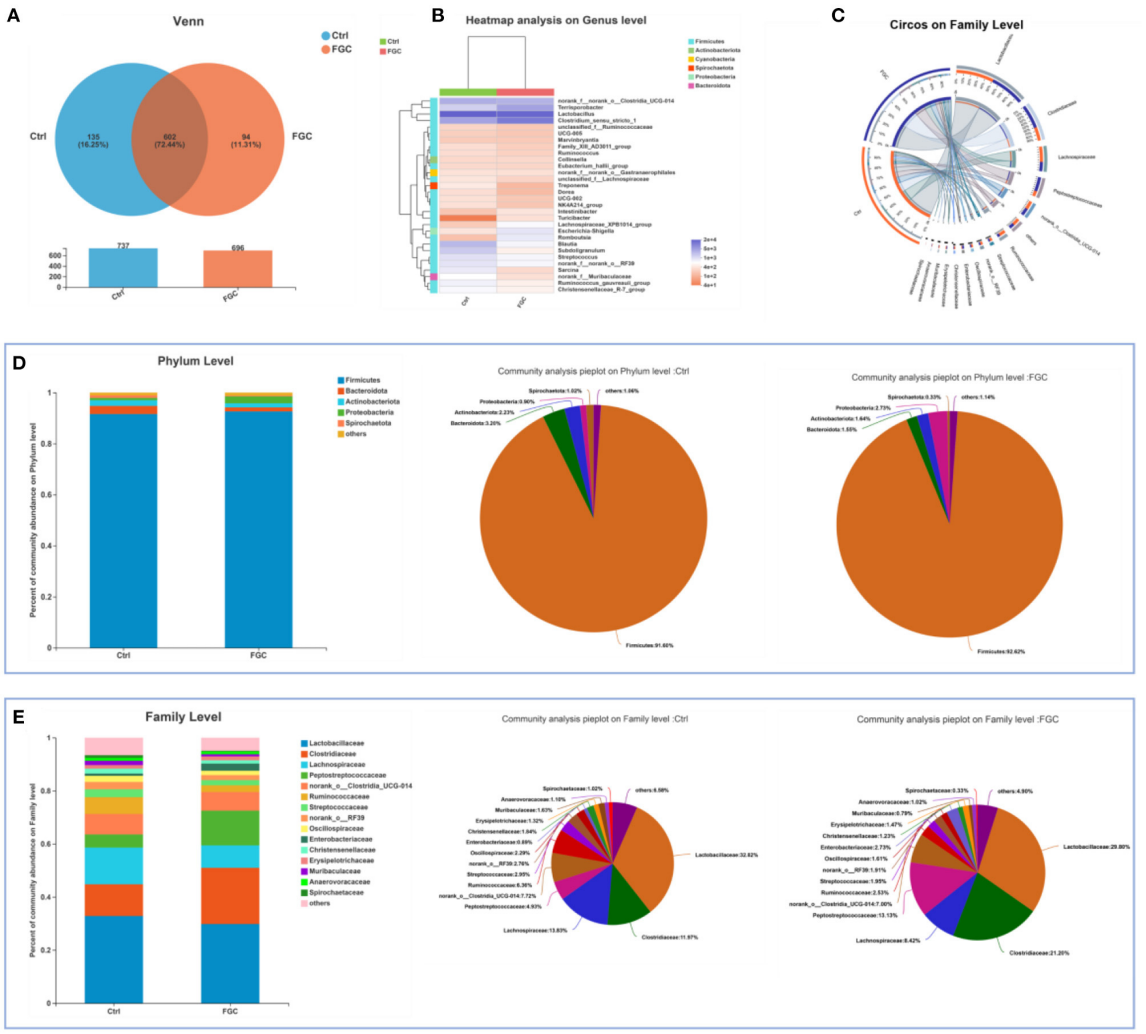
Overview of fecal microbial composition of 28d-piglets. **(A)** Venn diagram. **(B)** Heatmap at genus level. **(C)** Circos diagram at family level. **(D)** Barplot and pieplot at phylum level. **(E)** Barplot and pieplot diagram at family level. Ctrl, corn-soybean basal diet; FGC, corn-soybean basal diet with 2,000 mg/kg ferrous glycine chelate. *N* = 5.

For 14-day-old piglets, there were 227 and 62 unique OTUs and 508 common OTUs in the Ctrl and FGC from the Venn analysis (Figure 5A). At the phylum level, the dominant bacteria in piglet feces were primarily Firmicutes (91.90%), Bacteroidota (2.51%), Actinobacteriota (2.07%), and Spirochaetota (2.29%). After feeding FGC, the abundance of Firmicutes (86.73%), Actinobacteriota (0.55%), and Spirochaetota (0.23%) decreased and the abundance of Bacteroidota (10.38%) increased in piglet feces (Figures 5C,D). At the family level, the abundance of Clostridiaceae (16.23%), Muribaculaceae (8.94%), and Peptostreptococcaceae (6.75%) were enhanced and the abundance of Lactobacillaceae (26.32%), Streptococcaceae (1.05%), and Spirochaetaceae (0.23%) were lessened after feeding FGC (Figure 5E). At the genus level, the abundance of Clostridium_sensu_stricto_1 (16.21%), Blautia (13.80%), and norank_f_Muribaculaceae (8.94%) were increased and the abundance of Lactobacillus (26.32%) and Streptococcus (1.05%) were lessened after feeding FGC (Figure 5B). For 28 day-piglets, there were 135 and 94 unique OTUs and 602 common OTUs in the Ctrl and FGC from the Venn analysis (Figure 6A). At the phylum level, the dominant bacteria in piglet feces were primarily Firmicutes (91.60%), Bacteroidota (3.20%), Actinobacteriota (2.23%), Spirochaetota (1.02%), and Proteobacteria (0.90%). After feeding FGC, the abundance of Bacteroidota (1.55%) decreased and the abundance of Proteobacteria (2.73%) increased in piglet feces (Figures 6C,D). At the family level, the abundance of Clostridiaceae (21.20%%), Muribaculaceae (8.94%), and Peptostreptococcaceae (13.13%%) were enhanced and the abundance of Lachnospiraceae (8.42%%) and Spirochaetaceae (0.23%) were lessened after feeding FGC (Figure 6E). At the genus level, the abundance of *Clostridium_sensu_stricto_1* (20.51%), and *Terrisporobacter* (9.75%) were increased and the abundance of *Blautia* (1.99%) was lessened after feeding FGC (Figure 6B).

**Figure 7:**
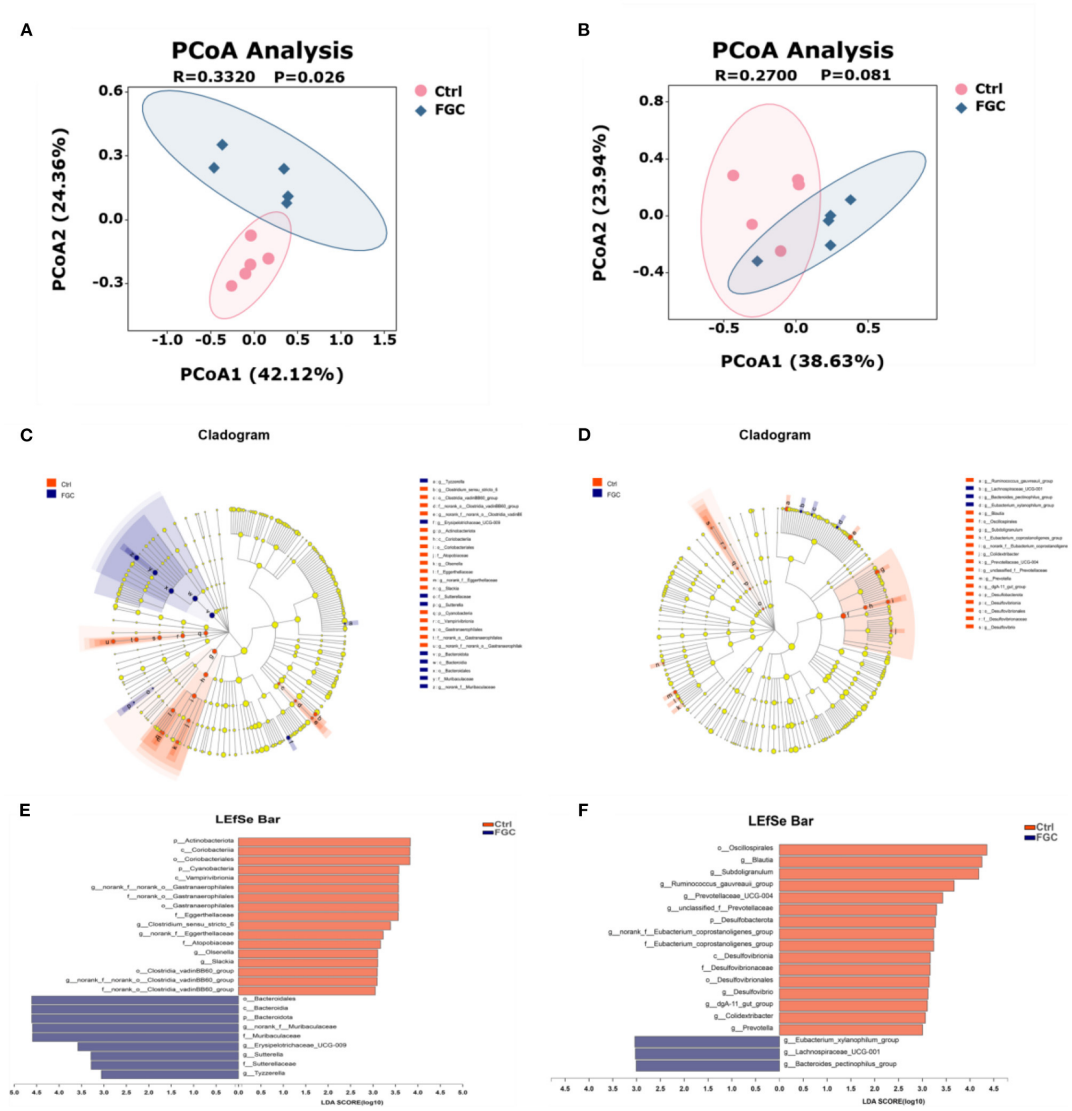
Fecal microbial β-diversity at OTU levels and LEfSe analysis from phylum to genus level of 14d and 28d-piglets. **(A)** PCoA of 14d-piglets 14. **(B)** PCoA of 28d-piglets. **(C)** Cladogram of 14d-piglets. **(D)** Cladogram of 28d-piglets. **(E)** LDA of 14d-piglets. **(F)** LDA of 28d-piglets. *p* < 0.05 and LDA score>3 were presented. LEfSe, linear discriminant analysis effect size; PCoA, principal co-ordinate analysis; LDA, linear discriminant analysis. Ctrl, corn-soybean basal diet; FGC, corn-soybean basal diet with 2,000 mg/kg ferrous glycine chelate. *N* = 5.

Samples were visualized using PCoA (Figures 7A,B) based on the Bray-Curtis distance algorithm, and differences between groups were examined with ANOSIM to evaluate the effect of FGC on the microbial structure of piglet feces. According to the PCoA results, dietary supplementation of FGC had a significant difference in fecal microorganisms for 14 day-piglets (*R* = 0.3320, *p* = 0.026). This difference, however, was not significant for 28 day-piglets (*R* = 0.2700, *p* = 0.081).

**Figure 8:**
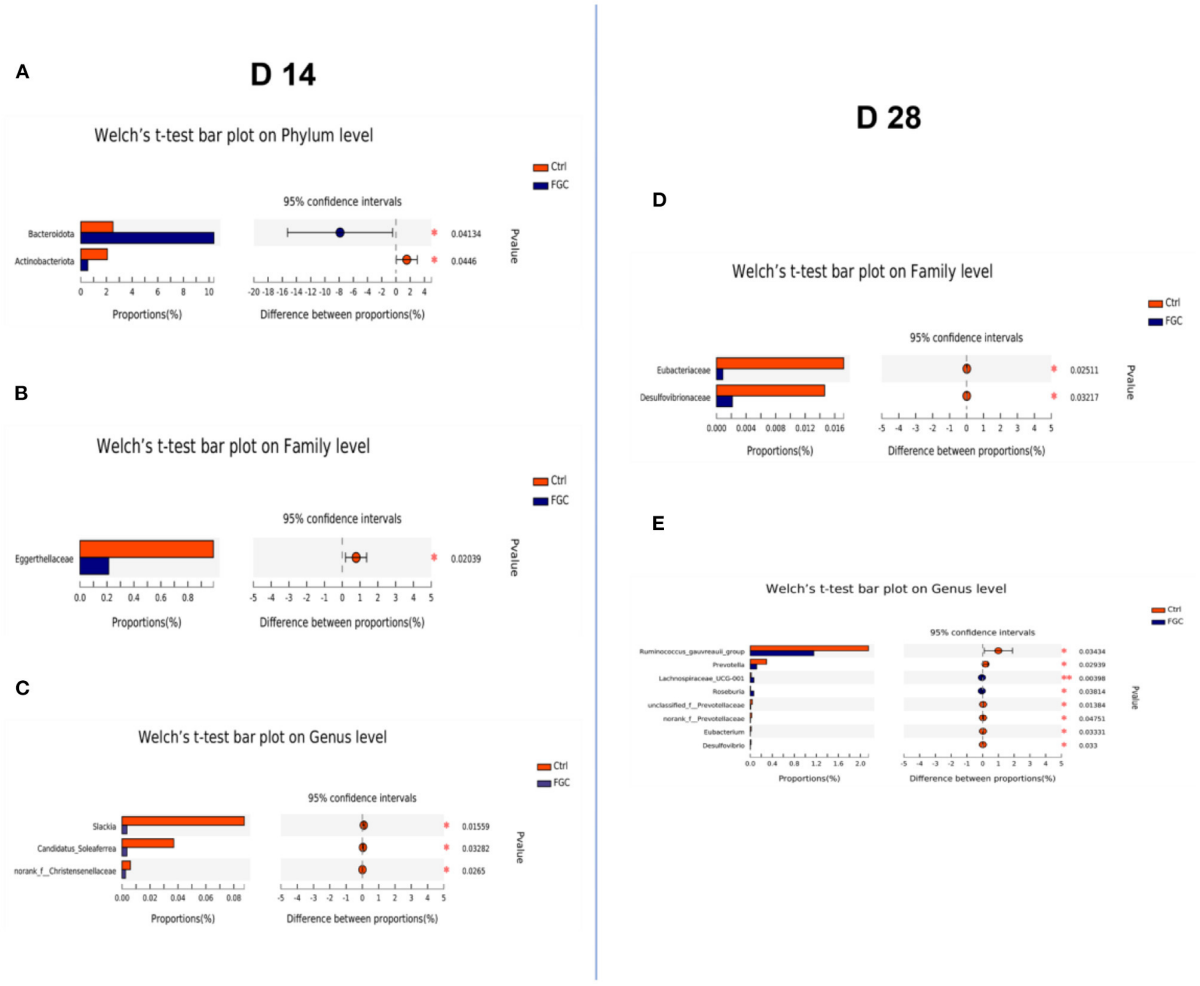
Histogram of fecal microbial community differences. **(A–C)** Differences in fecal microbiota of 14d-piglets at phylum, family, and genus levels. **(D,E)** Differences in the fecal microbiota of 28d-piglets at family and genus levels. Ctrl, corn-soybean basal diet; FGC, corn-soybean basal diet with 2,000 mg/kg ferrous glycine chelate. *N* = 5.

Besides, the results of LEfSe analysis (Figures 7C–F) revealed that the feces of piglets in the FGC group were significantly enriched (*p* < 0.05) in Bacteroidota (14 day), Muribaculaceae (14 day), *Erysipelotrichaceae_UCG-009* (14 day), *Sutterella* (14 day), *Lachnospiraceae_UCG_001* (14d), *Tyzzerella* (14 day), and *Bacteroides_pectinophilus group* (28d). Nevertheless, the relative proportions of bacteria such as *Olsenella* (14 day), *Slackia* (14 day), *Blautia* (28 day), *Ruminococcus_gauvreaui_group* (28 day), *Prevotella* (28 day), and *Desulfovibrio* (28 day) were significantly lower (*p* < 0.05) in the feces of FGC piglets. Welch's *t*-test with FDR correction was calculated to assess the significance of the difference from the observed sample (Figure 8), the FGC supplementation increased (*p* < 0.05) the relative abundance of Bacteroidota (14 day), *Lachnospiraceae_UCG_001* (28 day) and decreased (*p* < 0.05) the relative abundance of Actinobacteniota(14 day), Eggerthellaceae (14 day), Eubacteriaceae (28 day), Desulfovibrionaceae (28 day), *Slackia* (14 day), *Candidatus_Soleaferea* (14 day)*, Ruminococcus_gauvreaui_group* (28 day), and *Prevotella* (28 day).

### Correlation Analysis, Functional Prediction Analysis, and Volatile Fatty Acid

**Figure 9:**
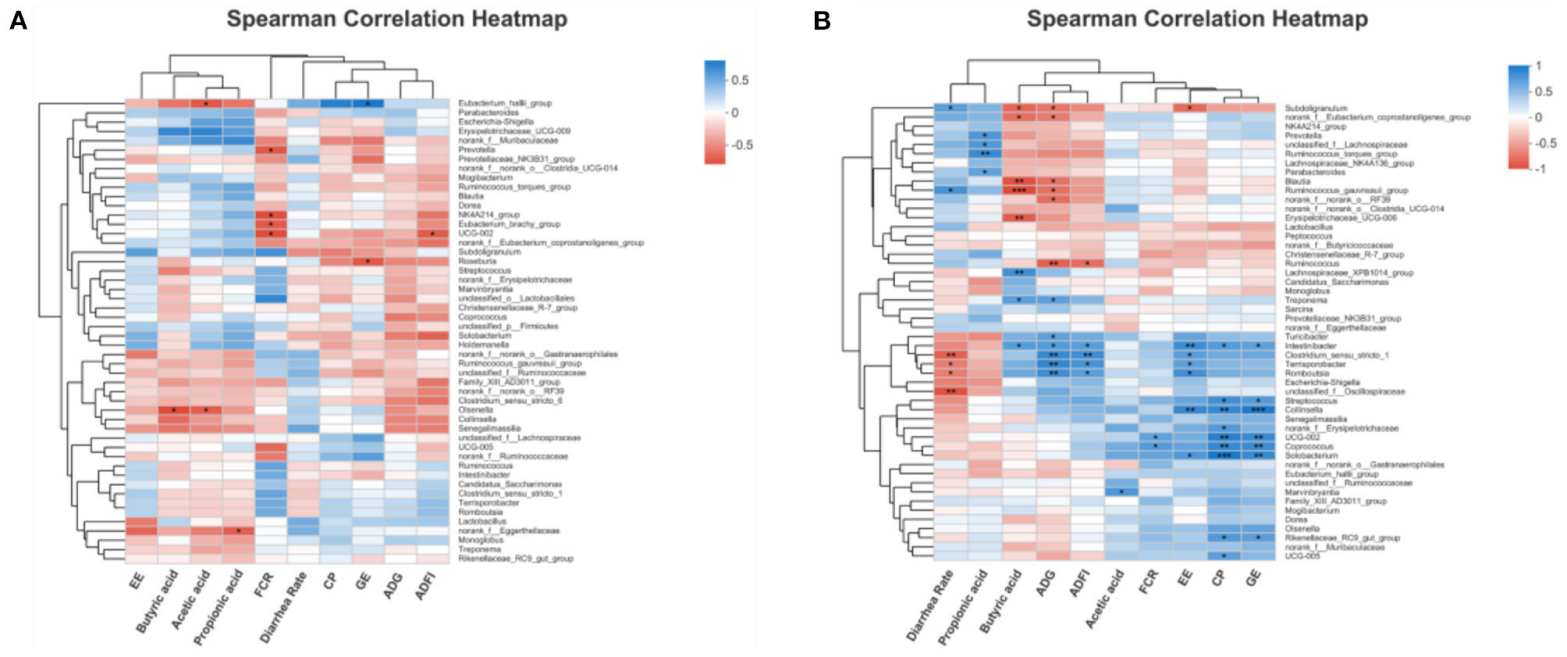
Correlation heatmap analysis among performance indices and significantly differential bacteria for **(A)** 14d-piglets and **(B)** 28d-piglets. ADG, average daily gain; ADFI, average daily feed intake; GE, gross energy; CP, crude protein; FCR, feed conversion ratio; EE, ether extract. Ctrl, corn-soybean basal diet; FGC, corn-soybean basal diet with 2,000 mg/kg ferrous glycine chelate. Red: positive correlation; blue, negative correlation. *p < 0.05; **p < 0.01; ***p < 0.001. *N* = 5.

The interaction between fecal microbial and growth characteristics as well as their metabolites of piglets was revealed using Spearman's correlation analysis. For 14day-piglets (Figure 9A), *Olsenella* correlated significantly and negatively (*p* < 0.05) with acetic acid and butyric acid, *Prevotella* showed a significant negative correlation (*p* < 0.05) with FCR. Likewise, for 28 day-piglets (Figure 9B) *Ruminococcus_gauvreaui_group* and *Blautia* exhibited a significant negative (*p* < 0.05) correlation with butyric acid and ADG. However, the microorganisms mentioned above were reduced in the feces of piglets supplemented with FGC.

**Figure 10:**
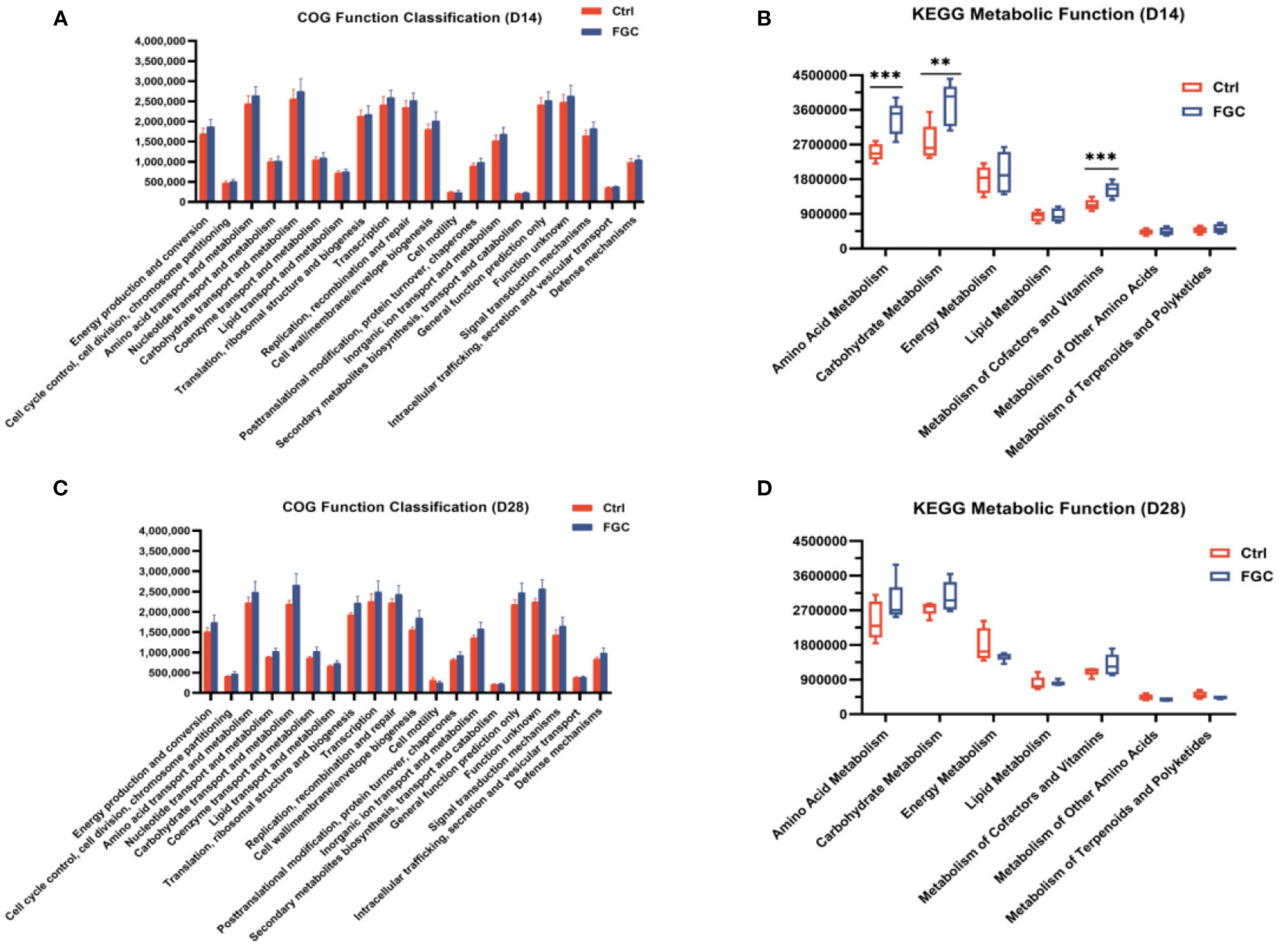
Predictive functional analysis of fecal microbiota in 14d and 28d-piglets. **(A)** COG function classification of 14d-piglets **(B)** KEGG metabolic function of 14d-piglets at pathway level 2. **(C)** COG function classification of 28d-piglets **(D)** KEGG metabolic function of 28d-piglets at pathway level 2. COG, Clusters of Orthologous Groups; KEGG, Kyoto Encyclopedia of Genes and Genomes. Ctrl, corn-soybean basal diet; FGC, corn-soybean basal diet with 2,000 mg/kg ferrous glycine chelate. Data were shown as means ± SEM. **p < 0.01; ***p < 0.001. *N* = 5.

Obtaining annotation information and abundance information of OTUs at each functional level of COG and KEGG (three level) based on the PICRUSt1 functional prediction model. As illustrated in Figure 10, for 14d-piglets, dietary supplementation of FGC enhanced the amino acid metabolism (*p* < 0.05), carbohydrate metabolism (*p* < 0.05), and metabolism of cofactors and vitamins (*p* < 0.05) on the pathway level 2 (Figure 10B), mainly impact the propanoate metabolism and butanoate metabolism on pathway level 3 (Supplementary Table S2). Nevertheless, no significant change in the metabolic function of KEGG for 28 day-piglets supplemented with FGC (Figure 10D, Supplementary Table S3).

**Figure 11:**
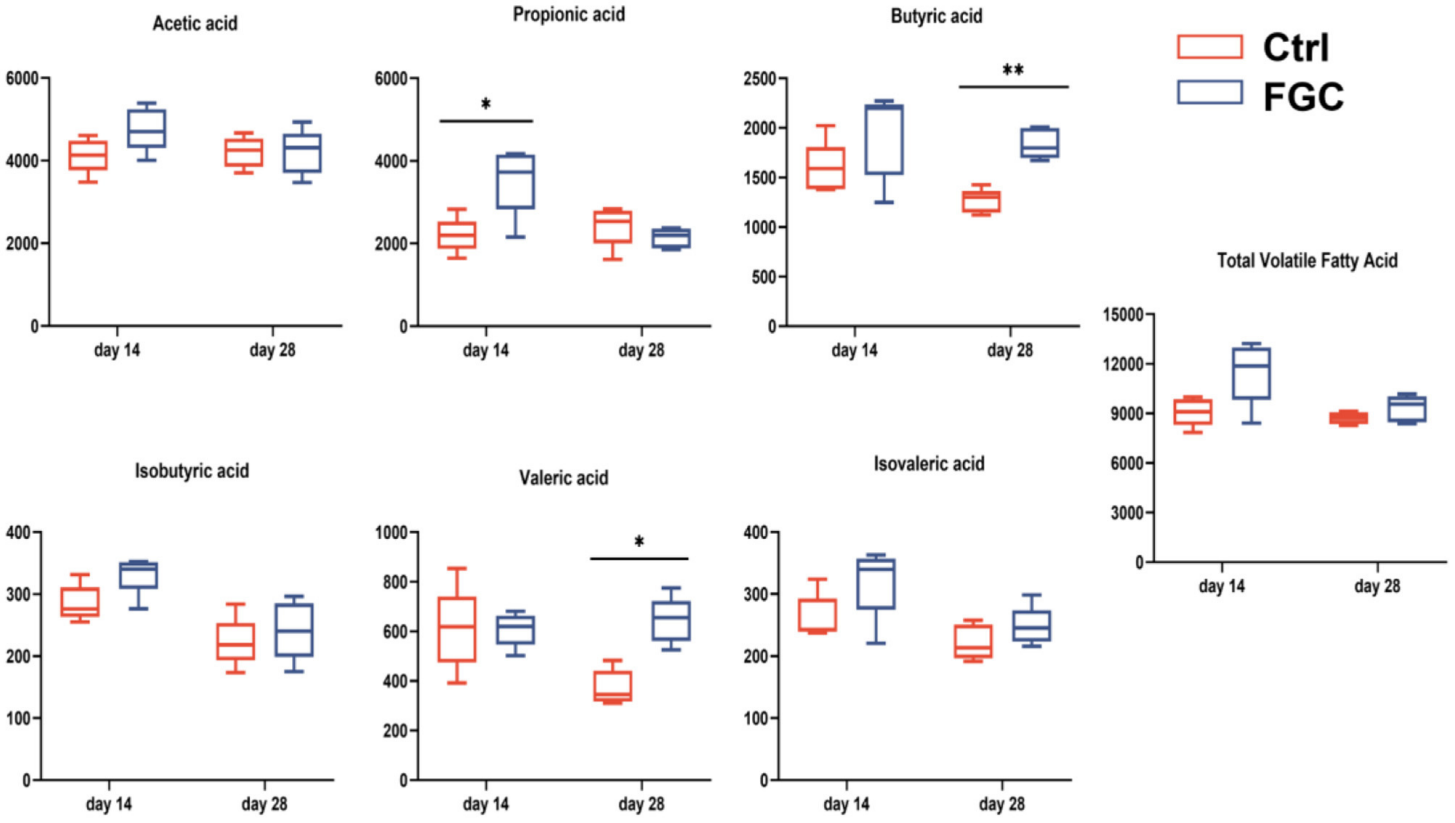
Fecal volatile fatty acid of piglets as affected by dietary FGC supplementation (mg/g). Ctrl, corn-soybean basal diet; FGC, corn-soybean basal diet with 2,000 mg/kg ferrous glycine chelate. Data were shown as means ± SEM. **p < 0.01; *p < 0.05. *N* = 5.

For fecal volatile fatty acid, 14 day-piglet supplementation with FGC increased the level of propionic acid (*p* < 0.05), and a higher level of butyric (*p* < 0.05) and valeric acids (*p* < 0.05) was observed in 28d-piglets. Nonetheless, no significant changes were noticed for the other volatile fatty acids between the Ctrl and FGC groups (Figure 11).

## Discussion

The weaning of piglets is typically accompanied by changes in the environment and diet, such as leaving the sow to start living in a group, switching from suckling to solid feed, which is a series of stress factors for weaned piglets (18). Moreover, piglets need a considerable amount of iron for their rapid growth rate, the increased blood volume, and the number of red blood cells, while piglets have fewer iron reserves in their bodies and insufficient iron is available in breastmilk to supply growth, thus tend to cause iron deficiency (19). Generally, the neonatal piglets used the strategy of extra iron injection to satisfy the iron requirement for piglet growth, while after weaning, dietary iron supplementation is a common way to prevent iron deficiency in weaned piglets (20, 21). In the current study, dietary FGC supplementation improved the ADG of piglets from day 0–7, day 15–28, and overall alleviated diarrhea in the pre-piglet period, which was consistent with the previous findings (22, 23). There are some reasons for this result. First, glycine is the smallest molecular weight of all essential amino acids, which contributes to the stability of the glycine ferrous chelate structure and reduces the interference of other iron inhibitors in the gastrointestinal tract, thus facilitating the absorption of iron (24, 25). Second, iron is indispensable activator of various enzymes involved in carbohydrate metabolisms, such as iron-containing cytochromes, cytochrome oxidase, catalase, and peroxidases. Ferrous glycinate, due to its good biological utilization, improves the content of iron-containing compounds and the enzymatic activity of iron-containing enzymes, which contributes to the carrier composition, transport, organismal substance metabolism, and energy metabolism, thus promoting piglets' performance (26).

Aiming to further investigate the effect of dietary FGC supplementation on piglet growth, the blood was collected from piglets at three stages (day 7, 14, and 28) and analyzed for relevant serological parameters including immunological characteristics, antioxidant capacity, and biochemical metabolic parameters. Notably, our results indicated that dietary FGC supplementation had no significant difference on the immune performance of piglets but increased the serum AKP and glucose levels and decreased the serum TC, LDL, and AST levels. Also, serum UA reduced but did not significantly different. AKP induces an increase in bone ratio and facilitates increased deposition of calcium and phosphorus in bone tissue (27). Elevated serum glucose levels indicated an increased level of carbohydrate metabolism, which was in line with the predicted results of the microbial KEGG functional model. The reduction of serum levels of TC, LDL, AST, and UA is beneficial to the healthy growth of piglets (28, 29).

Post-weaning piglets are stimulated by multiple factors such as environment and feed, causing disorders in the redox system of the organism and a large accumulation of free radicals in the piglets or reduced scavenging ability, which lead to slow growth and increased diarrhea, especially for smaller piglets (30). For the antioxidant system, iron is an indispensable activator of enzymes involved in redox reactions, such as SOD, CAT, and XOD. Iron deficiency or excessive could trigger the production of large amounts of free radicals, resulting in various diseases. SOD and CAT protect cells from the accumulation of H_2_O_2_, which prevents potential damage to cells by reactive oxygen ions (ROS) (31, 32). XOD is an essential source of free radicals in the body (33). MDA is an indicator reflecting the degree of tissue peroxidation. Previous research has indicated that exogenous iron supplementation increases SOD (34) and CAT (35) activities in rat liver and iron deprivation attenuates the XOD activity in male rats, which is in accordance with the results of the present study. Therefore, the improved growth performance and reduced diarrhea rate in piglets may be associated with improved antioxidant function. Furthermore, XOD mainly catalyzes the oxidation of hypoxanthine and xanthine to uric acid (36), which also corresponded to a decrease in serum UA levels.

Serum iron is primarily iron bound to transferrin in serum and is considered a marker of iron levels in the circulatory system. TIBC is the maximum amount of iron that can be bound by transferrin per liter of serum, which actually reflects the level of transferrin (37–39). Numerous studies have revealed that the total serum iron-binding capacity increased and serum iron and serum ferritin levels decreased when iron deficiency occurred in the body, and these indicators can be increased or decreased accordingly after iron supplementation (10, 40). Similarly, in the current study, dietary FGC supplementation attenuated the level of serum iron and decreased the level of TIBC, which indicated that FGC supplementation contributes to the transport and absorption of iron in piglets. Furthermore, a higher level of serum growth hormone and increased nutrient digestibility of ether extract and acid detergent fiber during the experiment was observed in the FGC group, which may help explain the improved growth performance and feed intake of piglets.

Undeniably, the intestinal microbiota plays a crucial role in the physiological and health status of the host (41). Age and feed are the principal factors affecting microbial colonization of the piglet gut (42, 43). The Chao and Ace indices indicated the community richness of microbiota and the Simpson index reflected the community diversity. The present study revealed that Firmicutes and Bacteroidetes were the two predominant phyla in the piglets, which is in agreement with previous studies on piglets (42, 44, 45). Additionally, dietary FGC supplementation enhanced the Simpson index and decreased the Ace and Chao indices at day 14, which suggested that both the community diversity and the community richness decreased. Such phenomenon illustrates that FGC supplementation interferes with or inhibits the proliferation of certain microorganisms in the gut of piglets and this intervention had no effect on the subsequent growth of the piglets. The PCoA results revealed that there was a significant difference in the fecal microbial structure of piglets between FGC and Ctrl at the early stages, and the difference became insignificant in the later stages.

Further analysis by LEfSe and Welch's test of microbiota community revealed that the abundance of bacteria such as *Olsenella, Prevotella*, and *Slackia* decreased and the abundance of microorganisms such as *Tezzerella* and *Sutterella* increased in FGC-fed piglets. *Slackia* was linked to pyometra (46). *Olsenella* was recognized as a microbiota involved in root canal infections associated with human teeth (47). *Prevotella*, as a potential pathogen, was associated with chronic inflammation (48). Likewise, *Olsenella*, and *Prevotella* were shown to have a significant negative correlation with piglet performance in the correlation analysis. *Tezzerella* was negatively correlated with LPS but positively correlated with short-chain fatty acids (49). Therefore, the increased abundance of *Tezzerella* favored short-chain fatty acid production, which also explained the increased levels of propanoate and butanoate metabolisms. The production of propionic and butyric acids facilitates the restoration and proliferation of intestinal epithelial cells, improves the intestinal barrier, and maintains the intestinal health of piglets (50). *Sutterella*, a pathogenic bacterium that could secrete IgA protease and degrades IgA (51), is one of the important sources of lipopolysaccharide. Moreover, studies have shown that *Sutterella* has a positive association with diarrhea (52), which may be the reason for the higher rate of diarrhea in piglets in the FGC group from day 8–14. It is currently unclear why the abundance of the pathogen *Sutterella* was increased in piglets fed FGC, which is probably because the production of short-chain fatty acids in the piglets' hindgut provides a suitable environment for *Sutterella* to proliferate (53). Therefore, it is necessary to study the optimal concentration of FGC to avoid the overproliferation of *Sutterella*.

## Conclusions

Briefly, dietary supplemented 2,000 mg/kg of FGC improves the performance and alleviates the diarrhea of piglets by enhancing antioxidant properties, improving iron transport, up-regulating the growth hormone, modulating the fecal microbiota as well as increasing the metabolism function. This improvement facilitates the transition of weaned piglets at the early stage. Hence, our next priority is to focus on the intestinal health of neonatal piglets and to determine the optimal FGC concentration to better address early iron deficiency in piglets.

## Data Availability Statement

The datasets presented in this study can be found in online repositories. The names of the repository/repositories and accession number(s) can be found in the article/Supplementary Material.

## Ethics Statement

The animal study was reviewed and approved by all procedure of current study were licensed by the Institutional Animal Care and Use Committee of China Agricultural University (No. AW10601202–1-2, Beijing, China).

## Author Contributions

JM and XP: conceptualization. JM, SL, XP, and JW: methodology. JM: software, investigation, data curation, and writing—original draft preparation. JM and SL: validation. JM, SL, and CW: formal analysis. Y-SL, T-PH, and LL: resources. XP, CW, Y-SL, T-PH, and LL: writing—review and editing. XP: supervision, project administration, and funding acquisition. All authors contributed to the article and approved the submitted version.

## Funding

This research was funded by the National Natural Science Foundation of China (31772612), the Beijing Municipal Natural Science Foundation (6202019), and the National Key Research and Development Program of China (2021YFD1300201).

## Conflict of Interest

Y-SL and T-PH were employed by Shanghai Bestar biochemical Co. Ltd. LL were employed by Tianjin Zhongsheng Feed Co. Ltd. The remaining authors declare that the research was conducted in the absence of any commercial or financial relationships that could be construed as a potential conflict of interest.

## Publisher's Note

All claims expressed in this article are solely those of the authors and do not necessarily represent those of their affiliated organizations, or those of the publisher, the editors and the reviewers. Any product that may be evaluated in this article, or claim that may be made by its manufacturer, is not guaranteed or endorsed by the publisher.
