# Supplementary material for: Dietary Supplementation of Ferrous Glycine Chelate Improves Growth Performance of Piglets by Enhancing Serum Immune Antioxidant Properties, Modulating Microbial Structure and Its Metabolic Function in the Early Stage
Source: Front Vet Sci. 2022 Apr 25;9:876965. doi: 10.3389/fvets.2022.876965 (PMC9083199; doi:10.3389/fvets.2022.876965)
Supplement: Supplementary file 1 [file Table_1.docx]

**Table S1 Composition and nutrient levels of basal diet (as-fed basis, %)**

| **Items** |  | **Items** |  | |  |
| --- | --- | --- | --- | --- | --- |
| **Ingredients** |  | **Nutritional levels (Calculated)** | | |  |
| Corn, 8.2% CP | 60.52 | Digestive energy, MJ/kg | | 14.74 | |
| Soybean meal, 46% CP | 16.00 | Crude protein | | 18.50 | |
| Extruded full-fat soybean meal, 35.6% CP | 7.00 | Calcium | | 0.74 | |
| Fish meal, 64.7% CP | 5.90 | Total phosphorus | | 0.62 | |
| Whey powder, 3.8% CP | 2.50 | Digestible lysine | | 1.22 | |
| Soybean oil | 4.86 | Digestible methionine | | 0.44 | |
| Dicalcium phosphate | 1.17 | Digestible threonine | | 0.72 | |
| Limestone | 0.18 | Digestible tryptophan | | 0.21 | |
| Salt | 0.25 | **Nutritional levels (Analyzed)** | | |  |
| L-lysine HCl, 78% | 0.37 | Gross energy, MJ/kg | | 17.58 | |
| L-methionine, 98% | 0.12 | Crude protein | | 18.82 | |
| Threonine, 98% | 0.13 | Ether extract | | 8.34 | |
| Tryptophan, 98% | 0.05 | Dry matter | | 88.49 | |
| Zinc oxide | 0.20 | Organic matter | | 94.97 | |
| Chromium trioxide | 0.25 | Neutral detergent fiber | | 13.19 | |
| Non-antibiotic premix^1^ | 0.50 | Acid detergent fiber | | 5.12 | |
| Total | 100.00 |  |  | |  |

^1^ Non-antibiotic premix for per kilogram diet included: vitamin A, 12,000 IU; vitamin D_3_, 2000 IU; vitamin E, 24 IU; vitamin K_3_, 2.0 mg; vitamin B_1_, 2.0 mg; riboflavin, 6.0 mg; vitamin B_6_, 3 mg; vitamin B_12_, 24 μg; nicotinic acid, 30 mg; pantothenic acid, 20 mg; folic acid, 3.6 mg; biotin, 0.1 mg; choline chloride, 0.4 mg; iron (from FeSO_4_·7H_2_O), 96 mg; copper (from CuSO_4_·5H_2_O), 8.0 mg; zinc (ZnSO_4_·H_2_O), 120 mg; manganese (MnSO_4_·H_2_O), 40 mg; iodine (from KI), 0.56 mg; selenium (from Na_2_SeO_3_), 0.4 mg.
