# Supplementary material for: Dietary Supplementation of Ferrous Glycine Chelate Improves Growth Performance of Piglets by Enhancing Serum Immune Antioxidant Properties, Modulating Microbial Structure and Its Metabolic Function in the Early Stage
Source: Front Vet Sci. 2022 Apr 25;9:876965. doi: 10.3389/fvets.2022.876965 (PMC9083199; doi:10.3389/fvets.2022.876965)
Supplement: Supplementary file 7 [file Image_1.pdf]

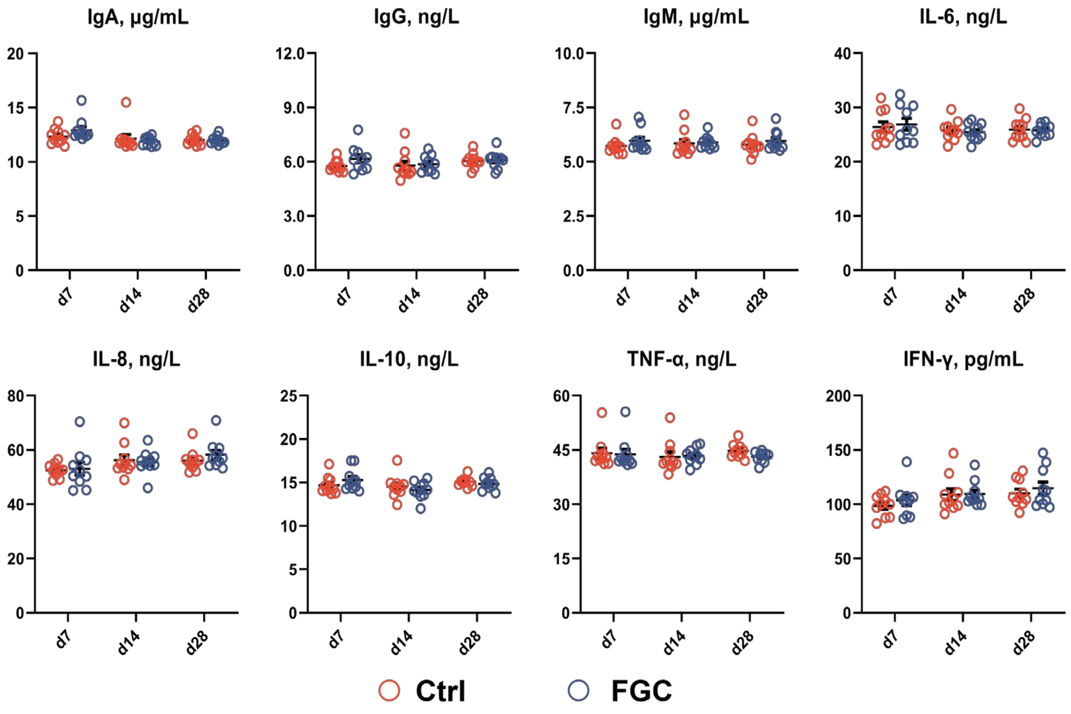

FIGURE S1| Serum immune and inflammatory factors of 7d, 14d and 28d-piglets as affected by dietary ferrous glycine chelate supplementation. IgA, G, M, immunoglobulin A, G, M; IL-6, 8, 10, interleukin 6, 8, 10; TNF-α, tumor necrosis factor-α, IFN-γ, gamma-interferon. Ctrl, corn-soybean basal diet; FGC, corn-soybean basal diet with 2,000 mg/kg ferrous glycine chelate. Data were shown as means ± SEM. N=10.
